# Pattern of childhood burn injuries and their management outcome at Bugando Medical Centre in Northwestern Tanzania

**DOI:** 10.1186/1756-0500-4-485

**Published:** 2011-11-09

**Authors:** Phillipo L Chalya, Joseph B Mabula, Ramesh M Dass, Geofrey Giiti, Alphonce B Chandika, Emmanuel S Kanumba, Japhet M Gilyoma

**Affiliations:** 1 Department of Surgery, Weill-Bugando University College of Health Sciences, Mwanza, Tanzania

**Keywords:** Childhood burn injuries, Patterns, Management outcome, Tanzania

## Abstract

**Background:**

Burn injuries constitute a major public health problem and are the leading cause of childhood morbidity and mortality worldwide. There is paucity of published data on childhood burn injuries in Tanzania, particularly the study area. This study was conducted to describe the pattern of childhood burn injuries in our local setting and to evaluate their management outcome.

**Methods:**

A cross sectional study was conducted at Bugando Medical Centre (in Northwestern Tanzania) over a 3-year period from January 2008 to December 2010. Data was collected using a pre-tested coded questionnaire and statistical analyses performed using SPSS software version 15.0.

**Results:**

A total of 342 burned children were studied. Males were mainly affected. Children aged = 2 were the majority accounting for 45.9% of cases. Intentional burn injuries due to child abuse were reported in 2.9% of cases. Scald was the most common type of burns (56.1%). The trunk was the most commonly involved body region (57.3%). Majority of patients (48.0%) sustained superficial burns. Eight (2.3%) patients were HIV positive. Most patients (89.8%) presented to the hospital later than 24 h. The rate of burn wound infection on admission and on 10th day were 32.4% and 39.8% respectively.*Staphylococcus aureus* were more common on admission wound swabs, with *Pseudomonas aeruginosa* becoming more evident after 10th day. MRSA was detected in 19.2% of *Staphylococcus aureus.* Conservative treatment was performed in 87.1% of cases. Surgical treatment mainly skin grafting (65.9%) was performed in 44 (12.9%) of patients. The overall average of the length of hospital stay (LOS) was 22.12 ± 16.62 days. Mortality rate was 11.7%. Using multivariate logistic regression analysis; age of the patient, type of burn, delayed presentation, clothing ignition, %TBSA and severity of burn were found to be significantly associated with LOS (*P* < 0.001), whereas mortality rate was found to be independently and significantly related to the age of the patient, type of burn, HIV positive with stigmata of AIDS, CD4 count, inhalation injury, %TBSA and severity of burn (*P* < 0.001).

**Conclusion:**

Childhood burn injuries still remain a menace in our environment with virtually unacceptable high morbidity and mortality. There is need for critical appraisal of the preventive measures and management principles currently being practiced.

## Background

Burn injuries constitute a major public health problem and are the leading cause of childhood morbidity and mortality worldwide [1-4]. The problem is even more conspicuous in a developing country like Tanzania where few specialized burn centres and trained burn professionals exist [5,6]. Burn injuries have been ranked as second most common cause of accidental death in children younger than 5 years and the most common cause accidental death in the home [1,7]. In Bugando Medical Centre, burn injuries in children are a common indication for paediatric surgical admission and contribute significantly to high morbidity and mortality.

Burns can be devastating injuries for children, the immediate effect of which is compounded by ongoing pain, cosmetic and physical disfigurement, impairment, multiple dressing changes and surgical procedures [8]. Burn injuries represent a stressful experience for burn victims as well as their family and the community in general [9]. Patients with extensive burns frequently die, and for those with less severe injuries, physical recovery is slow and painful [10]. In addition to physical damage caused by burns, patients also may suffer emotional and psychological problem [11].

Burns in children differ in multiple aspects from those in adults; the extent and depth of the burn injury are often more severe, the child's body proportions differ, resulting in greater evaporative water and heat loss, and fluid requirements are therefore generally greater. Children have a relatively thinner dermis, so for any given thermal insult the infant will sustain a deeper burn than the adult [12]. The pattern of burn injuries has been reported to vary from one community to another and is influenced by age, sex, economic status, local customs, social and environmental circumstances [13].

The management of paediatric burns and their sequalae remains demanding and extremely costly even in well-equipped, modern burn units of advanced affluent societies [9]. However, in most developing countries, late presentation to health facilities, lack of well equipped burn centres and trained medical personnel for treatment and rehabilitation of burn injury patients and non-existing early excision and skin grafting contributes significantly to increasing morbidity and mortality [14].

The outcome of burns is greatly influenced by the quality of care that patients receive. Inadequate public education, injury prevention and control measures coupled with absence of pre-hospital care and ineffective ambulance system for transportation of burned patients to specialized burn centers are important factors responsible for most of the deaths in cases of critical burns [4].

Since the most effective burns management is the prevention of burns accidents from occurring, it is of paramount importance to assess the current pattern of this health problem in our region with a view to identifying more preventive and therapeutic measures and ultimately improve the situation. This study was conducted in our local setting to describe our own experience in the management of childhood burn injuries, outlining the pattern and treatment outcome of these injuries with the hope that our findings will be a guide to offer preventive and therapeutic measures in these patients and ultimately improve their outcome.

## Patients and methods

### Study design and setting

A cross sectional study designed to describe the pattern of childhood burn injuries and their management outcome was conducted at the Accident & Emergency department and in the paediatric surgical wards of Bugando Medical Centre (BMC) over a 3-year period from January 2008 to December 2010. BMC is a 1000 bed referral hospital located in Mwanza city in Northwestern Tanzania on the southern border of Lake Victoria. It is also a teaching hospital for the Weill-Bugando University College of Health Sciences (WBUCHS). The hospital serves a population of approximately 13 million people from six regions in northwestern Tanzania (Mwanza, Mara, Kagera, Shinyanga, Kigoma and Tabora). More than 50% of this population seeks service from this hospital. Unfortunately the hospital has no dedicated burn unit for caring of burn patients as a result the vast majority of these patients are still managed in general surgical wards which are usually busy. Also, the hospital has no plastic/burn surgeons or specialist burn nurses. Patients with severe burn injuries are usually admitted in a multidisciplinary Intensive Care Unit (ICU) which also admits other medical and surgical patients. The criteria for admission to the hospital (paediatric surgical ward) according to Bugando Medical Centre protocol are that the patient has to have a burn of more than ten percent total body surface area (TBSA). However all burn injuries involving an inhalational component, electrical injuries, burns to face, hands, or the perineum, or circumferential burns will also be admitted, even if the TBSA is less than 10.0%

### Study subjects

The study included all burn injury patients aged 10 years and below presenting with acute burn injury and those who consented for the study and HIV testing. Patients admitted 7 days post-burn and patients admitted for post-burn reconstructive surgery were excluded from the study.

Recruitment of patients was done at the Accident and Emergency department and in the paediatric surgical wards after primary and secondary surveys done by the admitting surgical team. Patients who met the inclusion criteria were consecutively enrolled in the study after an informed written consent sought from the parents or guardians.

On admission, a sample of blood was taken from the patient and used to carry out HIV serology test and CD4+ cell count. HIV serology test was performed using the Tanzania HIV Rapid Test Algorithm [15]. CD4+ cell count was performed using FACS Count or FACSCALIBUR (BD Biosciences USA), to all HIV positive patients to determine the degree of immunosuppression. Polymerase Chain Reaction (PCR) was used to screen children under the age of 18 months. In addition, wound swabs were also taken for culture and sensitivity on admission and then on day ten after admission.

All recruited patients were managed according to advanced trauma life support [ATLS]. The%TBSA was assessed using a "Lund-Browder Chart". The burn depth was assessed clinically by observation of the burn wound. Patients with severe burns were subsequently admitted in the ICU and those with moderate burns were admitted to the paediatric surgical wards. Associated injuries were managed appropriately according to the type of injury. Patients were followed up until discharge or death. HIV positive patients were referred to the Care and Treatment Clinic (CTC) after post-counseling.

### Data collection

Data was collected using a pre-tested coded questionnaire. Data administered in the questionnaire included; Patient's characteristics (*i.e. age, sex, premorbid illness, HIV status*), circumstances of injury (*i.e. time and place of injury, nature of injury, type of burn, associated cloth ignition, associated injuries, inhalation injury, first aid measures at the scene and status of pre-hospital care)*, characteristic of burn wound *(i.e. burn depth, %TBSA, burn wound infection*), treatment parameters and outcome measures *(i.e. LOS, mortality, short-term complications*).

### Statistical analysis

Statistical analyses were performed using SPSS software version 15.0. Means, median, mode and standard deviation were used to summarize continuous variables whereas categorical variables were summarized in form of proportions and frequency tables. Data were categorized into categorical variables and Chi-square (¿ ^2^) test was used to test for significance of associations between the predictor and outcome variables in the categorical variables. Odds ratio (O.R.) was calculated to test for strength of association between predictor variables. Multivariate logistic regression analysis was used to determine predictor variables that are associated with outcome. Significance was defined as a p-value of less than 0.05.

### Ethical considerations

The study was carried out after the approval by the department of surgery and BMC/WBUCHS Ethics review board. An informed written consent was sought from the parents or guardians.

## Results

### Patient characteristics

During the period under study, a total of 342 burned children were studied. Males were 198 (57.9%) and females were 144 (42.1%) with a male to female ratio of 1.4:1 with a male predominance in each age group. Their ages ranged from 3 months to 10 years with a mean of 3.21 ± 2.42 years. The median and the mode were 3 and 2 years respectively. Children aged 2 years and below were the majority and accounted for 157 (45.9%) of cases. Table 1 shows distribution of patients according to age group and patient's gender. There was no significant difference between age group and patients' gender (*P *= 0.542).

**Table 1:** Distribution of patients according to age group and patients' gender

Age group/gender	Males	Females	Total
< 6	131 (38.3%)	97 (28.4%)	228 (66.7%)

6-10	67 (19.6%)	47 (13.7%)	114 (33.3%)

Total	198 (57.9%)	114 (42.1%)	342 (100%)

(¿^2 ^= 0.461, df = 1, *P*-value = 0.542)

Eight (2.3%) patients had premorbid illness namely epilepsy (3 patients), congenital heart disease (2 patients), sickle cell disease, pulmonary tuberculosis and recurrent UTI (1 patient each respectively). Eight (2.3%) patients were HIV positive. Of these, two (25.0%) patients had stigmata of AIDS. The mean CD4 count for HIV positive was 341 ± 226 cells/¿L. Three patients (37.5%) in the group of HIV infected patients had CD4 count ¿ 200 cells/¿L and the remaining 5 (62.5%) HIV positive patients had CD4 count > 200 cells//¿L.

### Circumstances of injury

Majority of burn injury, 310 (90.6%) occurred at home mainly in the kitchen, bathroom, sitting room and outdoors. Twenty-one (6.1%) patients sustained burns in the streets and in the remaining eleven (3.2%) patients the place of burn injury could not be established. The vast majority of burns, 332 (97.1%) were unintentional; intentional burns occurred in 10 (2.9%) patients mainly due child abuse. Most of burn injuries, 208 (60.8%) occurred during the day. Scald was the most common cause of burns, occurring in 192 (56.1%) of the patients (Table 2).

**Table 2:** Distribution of the type of burn by age

Age group (in years)	Type of burn					Total
		
	Scald	Flame	Chemical	Contact	Electrical	
0-2	114 (33.3%)	40 (11.7%)	1 (0.3%)	2 (0.6%)	-	157 (45.9%)

3-5	56 (16.4%)	35 (10.2%)	1 (0.3%)	1 (0.3%)	-	93 (27.2%)

6-10	22 (6.4%)	63 (18.4%)	3 (0.9%)	1 (0.3%)	3 (0.9%)	92 (26.9%)

Total	192 (56.1%)	138 (40.4%)	5 (1.5%)	4 (1.2%)	3 (0.9%)	342 (100%)

Patients who sustained scald burn had significant shorter LOS and low mortality rate than those who had other types of burn (*P *< 0.001).

Clothing ignition was reported in 38 (11.1%) patients who sustained flame burns. Patients who had associated clothing ignition had significant extensive and deep burns than those without clothing ignition (*P *= 0.002). Five patients (1.5%) had inhalation injury requiring intubation and ventilatory support. Of these, two patients had intubation and the remaining patients had only high flow nasal or mask oxygen. The overall mortality rate of the patients with inhalation injury was higher than those without inhalation injury (48.5% versus 5.7%). These differences were significant (*P *= 0.011).

Six patients (1.8%) in this study had associated injuries, of which fractures and head/neck injuries were the most common. Patients with associated injuries had significant higher mortality rate and prolonged LOS (> 14 days) than those without associated injuries (*P *< 0.001). Appropriate first aid measures at the site of accident (scene) were reported in 22 (6.4%) patients. There was significant association between proper first aid information and parents education (*P *= 0.011) and the place of residence (Urban and rural) (*P *= 0.013). First aid measures at the site of accident did not significantly influence both LOS (*P *= 0.213) and mortality (*P *= 0.951).

Only twenty-three (6.7%) of our patients had pre-hospital care. The vast majority of patients, 339 (99.1%) were brought in by parents and relatives. Only three (0.9%) patients were brought in by ambulance.

Most patients, 307 (89.8%) presented late (later than 24 h) to the to the A & E department with only 35 (10.2%) patients presenting within 24 h of burn. There was statistically significance difference between late presentation and LOS (*P *= 0.006) but not mortality (*P *= 0.0981).

The waiting time at the A & E department ranged from 10 min to six hours with a median of 2 h. The majority of patients, 302 (88.3%) were attended to within 2 h of arrival to the A & E department.

### Clinical characteristics of the burn wound

The trunk was the most commonly involved body region in 196 (57.3%) patients, followed by upper limbs in 142 (41.5%), lower limbs in 101 (29.2%) and head and neck in 75 (21.9%) patients. The genital and perineal areas were the least involved in 52 (15.2%). There was no significance difference between body regions burned and either LOS (*P *= 0.349) or mortality (*P *= 0.067).

The% TBSA ranged from 5% to 54% with the mean of 16.24 ± 7.42. The median and the mode were 14.00 and 15.00 respectively. A total of 322 (94.2%) patients had TBSA less than 40% and the remaining 20 (5.8%) patients had TBSA of 40% and above. Mortality in patients with%TBSA less than 40% was 8.6%, this increased to 42.5% in patients with%TBSA of 40% and above. These differences were significant (*P *= 0.012). The extent of burn (%TBSA) also significantly influenced the length of hospital stay (*P *= 0.010).

Majority of patients 164 (48.0%) sustained superficial burns only. The remaining patients had either deep burn 98(28.7%) or mixed burns 80(23.4%). Patients with superficial burns had significant short LOS and low mortality rate compared to patients with deep or mixed burns (*P *< 0.001). Majority of patients, 194 (56.7%) sustained moderate burns. Severe burns occurred in 148 (43.3%) patients. The severity of burn significantly influenced the length of hospital stay (*P *= 0.011) and mortality (*P *= 0.000).

The rate of burn wound infection on admission and on 10th day were 32.4% and 39.8% respectively. *Staphylococcus aureus *were more common on admission wound swabs, with *Pseudomonas aeruginosa *becoming more evident after 10th day. MRSA was detected in 19.2% of *Staphylococcus aureus*. Burn wound sepsis did not significantly influence both LOS (*P *= 0.457) or mortality (*P *= 0.271).

### Treatment parameters

Conservative treatment only with analgesics, antibiotics, tetanus toxiod, topical antimicrobial agents, antiseptics and wound dressing was performed in 298 (87.1%) patients. Surgical treatment was performed in 44 (12.9%) patients; of these 29 (65.9%) patients underwent skin grafting, 5 (11.4%) patients underwent fasciotomy and escharotomy, 4 (9.1%) patients underwent wound debridement and 3 patients underwent hip spica and finger amputation each respectively.

### Clinical outcome

The overall length of hospital stay (LOS) ranged from 1 to 86 days with a mean, median and mode of 22.12 ± 16.62, 18.00 and 1.00 days respectively. The length of hospital stay for non-survivors ranged from 1 to 16 days with the mean of 4.20 ± 2.80 days. The median was 2.00 days and the mode was 1.00 day signifying that most of deaths occurred within 24 h after admission. Table 3 shows the predictors of LOS according to univariate and multivariate logistic regression analysis.

**Table 3:** Predictors of prolonged LOS according to univariate and multivariate logistic regression analysis

Predictor variable	N (%)	Univariate analysis			Multivariate analysis		
		
		OR	**95% C.I**.	*P*-value	OR	**95% C.I**.	*P*-value
**Age(in years)**							

< 5	250 (73.1)						

5-10	92 (26.9)	3.85	(3.17-4.65)	0.011	2.87	(1.23-5.86)	**0.004**

**Sex**							

Males	198 (57.9)						

Females	144 (42.1)	1.82	(0.99-3.34)	0.056	2.12	(1.90-3.21)	0.177

**Pre-existing illness**							

Present	8 (2.3)						

Absent	334 (97.7)	2.29	(0.67-5.78)	0.088	0.26	(0.16-1.95)	0.456

**Timing of care**							

¿ 24 h	246 (71.9)						

> 24 h	96 (28.1)	14.43	(7.33-29.67)	0.002	3.54	(1.32-6.71)	**0.006**

**Type of burn**							

Scald	192 (56.1)						

Flame	138 (40.4)	1.21	(1.114-2.65)	0.032	1.61	(1.53-2.32)	**< 0.001**

Chemical	5 (1.5)	1.83	(0.81-4.11)	0.1	0.42	(0.21-0.95)	**< 0.001**

Electrical	3 (0.9	2.52	(1.32-3.81)	0.043	0.90	(0.93-0.98)	**< 0.001**

Contact	4 (1.2)	1.87	(1.01-2.69)	0.071	2.11	(1.03-2.42)	**< 0.001**

**Clothing ignition**							

Yes	38 (11.1						

No	304 (88.9)	2.21	(1.42-5.62)	0.023	1.73	(1.11-4.86)	**0.014**

**Associated injuries**							

Present	6 (1.8)						

Absent	336 (98.2)	9.34	(5.3-17.93)	0.003	1.71	(1.21-3.33)	**0.035**

**TBSA (%)**							

< 40	322 (94.2)						

¿ 40	20 (5.8)	2.38	(1.67-5.61)	0.002	2.97	(2.08-3.98)	**0.010**

**Inhalation injury**							

Present	5(1.5)						

Absent	337 (98.5)	1.06	(0.65-1.98)	0.038	0.27	(0.01-1.31)	0.058

**Burn depth**							

Superficial	164 (48.0)						

Deep	98(28.7)	0.32	(0.18-2.12)	0.953	0.21	(0.11-.3.91)	0.897

Mixed	80(23.4	1.94	(0.53-2.63)	0.212	1.85	(1.13-2.11)	0.065

**Severity**							

Moderate	194 (56.7)						

Severe	148(43.3)	0.26	(0.11-0.93)	0.001	4.32	(2.65-8.39)	**0.011**

**HIV status**							

Positive	8 (2.3)						

Negative	334 (97.7)	0.56	(0.21-1.93)	0.111	2.09	(1.23-2.98)	0.981

**CD 4 + count**							

< 200	3 (37.5)						

¿200	5(62.5)	0.93	(0.52-1.99)	0.123	1.33	(0.54-2.23)	0.072

Keys: TBSA = total body surface area, O.R. = Odds ratio, C.I. = Confidence Interval

Of the 342 patients, 40 died with an overall mortality rate of 11.7%. Table 4 shows the predictors of mortality according to univariate and multivariate logistic regression analysis.

**Table 4:** Predictors of mortality according to univariate and multivariate logistic regression analysis

Predictor variable	N (%)	Univariate analysis			Multivariate analysis		
		
		OR	**95% C.I**.	*P*-value	OR	**95% C.I**.	*P*-value
**Age(in years)**							

< 5	250 (73.1)						

5-10	92 (26.9)	2.67	(1.41-5.66)	0.021	4.61	(2.98-8.87)	**0.001**

**Sex**							

Males	198 (57.9)						

Females	144 (42.1)	0.43	(0.12-1.11)	0.065	0.23	(0.11-1.33)	0.431

**Pre-existing illness**							

Present	8 (2.3)						

Absent	334 (97.7)	1.22	(0.91-2.12)	0.025	1.34	(0.12-1.45)	0.056

**Timing of care**							

¿ 24 h	246 (71.9)						

> 24 h	96 (28.1)	1.98	(0.77-2.11)	0.048	0.96	(0.81-1.23)	0.098

**Type of burn**							

Scald	192 (56.1)						

Flame	138 (40.4)	1.76	(1.11-2.01)	0.112	0.04	(0.01-0.11)	**< 0.001**

Chemical	5 (1.5)	1.99	(1.13-2.11)	0.687	1.21	(1.10-2.09)	**< 0.001**

Electrical	3 (0.9	0.19	(0.03-0.83)	0.153	0.23	(0.11-0.56)	**< 0.001**

Contact	4 (1.2)	2.31	(2.01-2.89)	0.061	1.26	(1.06-1.51)	**< 0.001**

**Clothing ignition**							

Yes	38 (11.1						

No	304 (88.9)	0.04	(0.01-0.68)	0.032	0.91	(0.11-1, 76)	0.086

**Associated injuries**							

Present	6 (1.8)						

Absent	336 (98.2)	4.87	(3.44-8.92)	0.011	4.77	(2.81-8.94)	**0.000**

**TBSA (%)**							

< 40	322 (94.2)						

¿ 40	20 (5.8)	3.90	(2.93-6.93)	0.002	2.54	(1.69-7.44)	**0.012**

**Inhalation injury**							

Present	5(1.5)						

Absent	337 (98.5)	4.76	(2.09-7.77)	0.000	6.43	(4.21-9.39)	**0.011**

**Burn depth**							

Superficial	164 (48.0)						

Deep	98(28.7)	0.28	(0.11-1.21)	0.043	2.12	(0, 86-2.64)	0.098

Mixed	80(23.4	1.03	(0.83-1.75)	0.062	0.08	(0.02-1.21)	0.564

**Severity**							

Moderate	194 (56.7)						

Severe	148 43.3)	3.89	(2.11-7.93)	0.002	6.86	2.43-8.96)	**0.000**

**HIV status**							

Positive	8 (2.3)						

Negative	334 (97.7)	1.82	(0.22-1.99)	0.045	0.71	(0.12-1.99)	0.087

**Stigmata of AIDS**							

Present	2(25.0%)						

Absent	6 (75.0%)	2.87	(1.92-6.66)	0.012	3.34	(1.83-4.76)	**0.004**

**CD 4 + count**							

< 200	3 (37.5)						

¿200	5(62.5)	1.34	(1, 11-4.78)	0.003	2.98	(2.06-5.98)	**0.001**

## Discussion

Burn injuries in children continue to be a major public health problem responsible for significant morbidity and mortality at Bugando Medical Centre. In agreement with other studies [5-7,16,17], the majority of patients in this study were aged 2 years and below. High incidence of burn injuries in children reflects lack of coordination and unawareness of dangerous substances in this age group. In addition, poor supervision because of large families and lack of domestic safety measures play important role in occurrence of burn.

In our study, males were slightly more affected than females with a male to female ratio of 1.4:1 which is in agreement with other studies [6,7,18]. The reasons for the male preponderance in our study may be attributed to the overactive nature of male babies as compared to the females.

The presence of pre-existing illness has an impact on the outcome of burn injury [19]. In agreement with other studies [19,20], epilepsy was found to be the most common pre-existing illness in this study making these patients a special group which needs special care. In most cases epileptic patients sustain burn injury during epileptic attack. Therefore care must be taken to prevent them from burn injury.

In this study, intentional burn injuries mainly due to child abuse were reported in only 2.9% of cases. These figures were significantly low compared to what was reported in Uganda (16%) by Nakitto & Lett [21]. In comparison with accidentally burned children, abused children are significantly younger and have longer hospital stays and higher mortality rates [22]. The low figures of intentional burn injuries in our study may actually be an underestimate and the magnitude of the problem may not be apparent because many cases are not reported for fear of been arrested by police. Therefore, paediatric forensic examination should be performed if a child is likely to suffer from abuse, neglect or intentional injury.

The majority of burn injury in this study occurred at home mainly in the kitchen, which is in agreement with other studies done elsewhere [23-25]. The home remains a dangerous place for children as lack of enough space for children to play, during cooking and, storage of the hot fluids e.g. tea, porridge and water with uncovered containers in the single room may result in burn injury of the children who can easily reach the hot stuff.

In this review, scald was the main type of burn among children that agrees with other studies [19,26,27]. This may be related to densely populated families, physical environment of houses, child neglect, and child's inclination for touching things. In the present study, patients who sustained scald had significant shorter LOS and low mortality rate than those who had other types of burn. This can be explained by the fact that scald injury causes superficial burns which heal fast with no surgical intervention and therefore these patients have short hospital stay and low mortality rate compared to patients with deep or mixed.

First aid measures at the site of accident play a vital role in determining the final outcome of treatment when done appropriately. It contributes significantly to reducing morbidity and mortality. In this study, appropriate first aid measures at the site of accident were reported in only 6.4% of our patients. Similar low incidence of appropriate first aid measures among burned patients were noted in other studies [7,28]. However, Ramcharan *et al*. [29] in North Trinidad reported a high figure (65.1%) of patients performed first aid adequately at the site of accident. This discrepancy may be attributed to difference in public awareness and knowledge about first aid procedures for burns from one country to another. There was significant association between proper first aid information and parents education and the place of residence (Urban and rural). Although first aid measures at the site of accident did not significantly influence both length of hospital stay and mortality, we still believe that they play a vital role in determining the final outcome of treatment when done appropriately.

The prehospital care of burned patients is the most important factor in determining the ultimate outcome after burn injury [30]. In our study, only 6.7% of patients had pre-hospital care. The lack of advanced pre-hospital care in most developing countries like Tanzania and ineffective ambulance system for transportation of patients to hospitals are a major challenges in providing care for burn injury patients in these countries and have contributed significantly to poor outcome of these patients due to delay in definitive treatment.

Late presentation is the norm for most clinical conditions in our environment and burn injury is no exception. In this study, most patients (89.8%) presented late to the hospital with only 10.2% patients presenting within 24 h of burn. Late presentation in the present study may be attributed to delay in referral from private and public clinics, dispensaries and health centers, self-treatment at home, consultation with traditional healers and transport costs. Delayed presentation following burn injury increases the likelihood of death as well as prolonged hospital stay as the child may only be brought to hospital once the wound has become infected. Delay also results in deeper wounds and increased healing time.

The body region distribution trend in this study is consistence with other reports [20,31,32]. Although body region burn burned did not significantly influence both length of hospital stay and mortality, the authors still believe that body region burn burned has an influence on the outcome of burn care as it may result in functional or cosmetic impairment.

In this study, the vast majority of patients had TBSA burn of less than 40% which is in keeping with other reports [32-34]. The ultimate outcome of burn injury is influenced by the extent of burn (%TBSA) as shown by the present study.

Our bacterial profile trend was similar to that reported by others [20,31]. In Zaria, Nigeria it was found that *Pseudomonas aeruginosa *was the commonest isolated bacteria [16]. This difference in bacterial pattern reflects environmental differences in study setting. However, variations in antibiotics, tropical antimicrobial agents and dressing methods in the present study might have interfered with our results. The present study showed no significant difference between burn wound sepsis and the outcome of burn injury patients. Despite the above observations, the authors of the present study still believe that burn wound sepsis still contributes significantly to high morbidity and mortality among burn injury patients.

Our figures for HIV infection among paediatric burn injury patients (2.3%) in the present study was found to be significantly low than that reported in Malawi (5.8%) at the same age group reflecting differences in the overall prevalence of HIV infection in general population from one country to another [35]. HIV infection in children in these studies is most likely to be caused by vertical transmission or by blood transfusion for malaria-related anemia. Our study showed no significant difference in the outcome of HIV positive burn injury patients without stigmata of AIDS or those with CD4 count > 200 cells//¿L and HIV negative burn injury patients in terms of length of hospital stay and mortality. Similar observation was also noted in South Africa [36]. This implies that the prognosis of HIV positive burn injury patients depends mainly on the presence or absence of stigmata of AIDS and not on the presence of HIV antibodies.

Most of patients in this study were managed conservatively. Surgical treatment mainly skin grafting was performed in 12.9% of patients though more patients could have benefited from surgery. Similar observation was also reported by previous studies in Uganda [20,31] and North Trinidad [19]. There was no obvious explanation for the low incidence of surgical procedures in these series. Early excision and skin grafting in the management of deep burns have been reported to reduce infective complications, reduce mortality, shorten hospital stay and improve functional and aesthetic outcome [37]. In this study early excision and skin grafting was not usually practiced due to the fact that the majority of patients reported to the hospital late when they had already developed severe burn wound sepsis which needed dressing for a number of days before skin grafting. Lack of facilities and specialized professionals can also explain the low incidence of early excision and skin grafting in this study.

The overall mean LOS in this study was relatively higher compared to that reported in Saudi Arabia [13], but lower than in the Turkish study [4]. High figures for the LOS in our study is attributed to delayed presentation to health facilities following burn injury as a result the majority of patients reported to the hospital late when they had already developed severe burn wound sepsis which needed dressing for a number of days. Burn patients generally experience long hospital stays, and the accurate prediction of the length of those stays has strong implications for healthcare resource management and service delivery. Therefore, burns in childhood cause huge financial and social burdens on individuals, families, society and the nation. To reduce this burden, a burn prevention strategy and prevention program for the country should be developed.

The overall mortality figure (11.7%) in our study is closer to that reported in Uganda [20]. High mortality rate in the present study may be attributed to several factors. First, BMC being a referral hospital, it receives many patients with large burns that are at high risk of death despite aggressive treatment. Second, Bugando Medical Centre has no burn unit as a result majority of patients are still admitted and managed in general surgical wards which are not well equipped in managing burn injury patients. Third, early excision and skin grafting in the management of burn is not adopted at Bugando Medical Centre.

The potential limitations in this study is that it only reports on children admitted for burns to one major tertiary hospital may not be truly representative for the general population. Also, nutritional status which is a known factor influencing the outcome of burn injury patients was not measured due to failure to get pre-burn weight which would be used to measure body mass index and weight for age. Variations in antibiotics, topical antimicrobial agents and dressing methods might have affected the results.

## Conclusion

This study shows that childhood burn injuries continue to be a challenging problem in our setting due to poor medical facilities, lack of well equipped burn centres and specialized professionals and absence of public awareness. Children aged two years and below are commonly affected. Most of these injuries occur at home and scald injuries predominate. Prehospital interventions in our environment are mostly deleterious and late presentation to health faculties following burn injury is a common phenomenon and it is associated with prolonged hospital stay resulting in increased costs of care as well as consumption of hospital resources. Appropriate and timely treatment of these patients can ensure excellent outcome in majority of cases. We recommend that there is need for more public health enlightenment on the prevention and initial intervention in burns in children. Measures targeting at establishment of burn centres in our environment is of paramount.

## Competing interests

The authors declare that they have no competing interests.

## Authors' contributions

PLC conceived the study and did the literature search, coordinated the write-up, participated in data analysis, editing and submission of the article. JBM, RMD, GG, ABC and ESK participated in the literature search, writing of the manuscript and editing. JMG coordinated the write-up, editing and supervised the study. All the authors read and approved the final manuscript.

## References

[B1] Ryan CA Shankowsky HA Tredget EE Profile of the paediatric burn patient in Canadian burn centre Burns 1992 18 267 272 10.1016/0305-4179(92)90146-L 1418501

[B2] Ahuja RB Bhattacharya S Burns in the developing world and burn disasters Br Med J 2004 329 447 449 10.1136/bmj.329.7463.447 PMC514214 15321905

[B3] Mashreky SR Rahman A Chowdhury SM Epidemiology of childhood burn: yield of largest community based injury survey in Bangladesh Burns 2008 34 856 862 10.1016/j.burns.2007.09.009 18242869

[B4] Aysun BO Ali RT Alper K Kaya Y Burn injuries among children aged up to seven years Turk J Pediatr 2009 51 328 335 19950839

[B5] Mbaga FWM Mwafongo VG A profile of burn injury in Dar es Salaam, Tanzania Tanzan Med J 1998 3 2 8 12

[B6] Temu MJ Rimoy G Premji Z Matemu G Causes, magnitude and management of burns in underfives in district hospitals in Dar Es Salaam, Tanzania East Afr J Public Health 2008 5 1 38 42 18669122 10.4314/eajph.v5i1.38975

[B7] Okoro PE Igwe PO Ukachukwu AK Childhood burns in south eastern Nigeria Afr J Paediatr Surg 2009 6 24 27 10.4103/0189-6725.48571 19661661

[B8] Mukerji G Chamania S Patidar GP Gupta S Epidemiology of paediatric burns in Indore, India Burns 2009 27 33 38 10.1016/s0305-4179(00)00058-9 11164662

[B9] Atiyeh BS Costagliola M Hayek SN Burn prevention mechanisms and outcomes: pitfalls, failures and successes Burns 2009 35 2 181 193 10.1016/j.burns.2008.06.002 18926639

[B10] Benito-Ruiz J An analysis of burn mortality; a report from a Spanish regional burn centre Burns 1991 17 201 204 10.1016/0305-4179(91)90104-O 1892551

[B11] Army BN Walter NJ Gregory BM Graves G Psychological effect of burn injuries J Am Assoc 1998 253 245 254

[B12] Lowell G Quinlan K Gottlieb LJ Preventing unintentional scald burns: Moving beyond tap water Paediatrics 2008 122 799 804 10.1542/peds.2007-2979 18829804

[B13] Al-Shehri M The pattern of paediatric burn injuries in Southwestern Saudi Arabia West Afr J Med 2008 23 4 294 299 10.4314/wajm.v23i4.28144 15730087

[B14] Dongo AE Irekpita EE Oseghali LO Ogbebor CE Iyamu CE Onuminya JE A five-year review of burn injuries in Irrua BMC Health Serv Res 2007 7 171 10.1186/1472-6963-7-171 17956614 PMC2174937

[B15] Lyamuya EF Aboud S Urassa WK Sufi J Mbwana J Ndungulile F Massambu C Evaluation of rapid HIV assays and development of National Rapid HIV test algorithms in Dar es Salaam, Tanzania BMC Infect Dis 2009 9 19 10.1186/1471-2334-9-19 19226452 PMC2650699

[B16] Kalayi GD Burn Injuries in Zaria. A one-year prospective study East Afr Med J 1994 71 5 317 321 7925065

[B17] Archbong AE Antia UE Udosen J Childhood burns in South Eastern Nigeria East Afr Med J 1997 74 6 382 384 9487401

[B18] Oludiran OO Umebese P Pattern and outcome of children admitted for burns in Benin City, mid-western Nigeria Ind J Plast Surg 2009 42 189 193 10.4103/0970-0358.59277 PMC2845362 20368855

[B19] Muller MJ Pegg SP Rule MR Determinants of death following burn injury Br J Surg 2001 88 583 587 10.1046/j.1365-2168.2001.01726.x 11298629

[B20] Chalya PL Factors affecting the outcome of burn injury patients admitted to Mulago Hospital, Kampala. Uganda Dissertation for Master of Medicine in Surgery, Makerere Medical School, Uganda 2006 42 54

[B21] Nakitto M Lett R Paediatric burn injuries: a hospital based study in Uganda Inj Prev 2010 16 A46 A47

[B22] Toon MH Maybauer DM Lisa L Arceneaux LL Frasera JF Meyere W Runge A Maybauer MO Children with burn injuries-assessment of trauma, neglect, violence and abuse J Inj Violence Res 2011 in press 10.5249/jivr.v3i2.91 PMC3134932 21498973

[B23] Feldman KW Schaller RT Feldman JA McMillan M Tap water scald burns in children Pediatrics 1998 102 1 256 258 683765

[B24] Sintha D Sie AMC Van-Rossum A Oudesluys M Scald Burn in the Bathroom: Accidental or Inflicted? Pediatrics 2004 113 1 173 174 10.1542/peds.113.1.173 14702475

[B25] Drago DA Kitchen scalds and thermal burns in children five years and younger Pediatrics 2005 115 1 10 16 15629975 10.1542/peds.2004-0249

[B26] Duggan D Quine S Burn injuries and characteristics of burn patients in New South Wales, Australia Burns 1995 21 83 89 10.1016/0305-4179(95)92129-Z 7766331

[B27] Rossi LA Braga EC Barruffini RC Carvalho EC Childhood burn injuries: circumstances of occurrences and their prevention in Ribeirao Preto, Brazil Burns 1998 24 416 9 10.1016/S0305-4179(98)00046-1 9725680

[B28] Kidanu EN Bernt L Epidemiology of burn injuries in Kelele Town Northern Ethiopia: a community based study J Health Dev 2002 16 1 1 7

[B29] Ramcharan R Das S Romany S Mohammed F Ali T Ragbir M Epidemiology of adult burns in North Trinidad Internet J Caribb Third World Med 2003 1 1

[B30] Mojgan K Moossa Z Mohammad RZ Ali K Childhood injuries in Tehran: a review of 1281 cases Turk J Pediatr 2008 50 317 325 19014043

[B31] Kakande I Thermal injuries in Mulago Hospital, Kampala East Afr Med J 1978 55 236 241 699831

[B32] Zaidi MM Abusselta A Brogowski K Agrawal PL Franka MR Analysis of burned children treated in Burns and Plastic Surgery Centre, Tripoly, Libya Ann Mediterr Burns Club 1993 4 45 47

[B33] Mzezewa S Jonson K Alberg M Salemrk L A prospective study on the epidemiology of burns in patients admitted to the Harare burn unit Burns 1990 25 499 504 10.1016/s0305-4179(99)00041-8 10498357

[B34] Chien W Pai L Lin C Epidemiology of hospitalized burns patients in Taiwan Burns 2003 29 589 591 10.1016/S0305-4179(03)00151-7 12927984

[B35] James J Hofland HW Borgstein ES Kumiponjera D Komolafe OO Zijlstra EE The prevalence of HIV infection among burn patients in a burn unit in Malawi and its influence on outcome Burns 2003 29 55 60 10.1016/S0305-4179(02)00236-X 12543046

[B36] Edge JM Vander-Merwe AE Pieper CH Bouic P Clinical outcome of HIV positive patients with moderate to severe burns Burns 2001 27 111 114 10.1016/S0305-4179(00)00090-5 11226644

[B37] Orgill DP Excision and Skin Grafting of Thermal Burns N Engl J Med of Medicine 2009 360 93 901 10.1056/NEJMct0804451 19246361

